# Resilience and caregiving ability among caregivers of people with stroke: The mediating role of uncertainty in illness

**DOI:** 10.3389/fpsyt.2022.788737

**Published:** 2022-11-22

**Authors:** Jinyao Wang, Jun Cui, Shuangyan Tu, Rong Yang, Lihong Zhao

**Affiliations:** ^1^Department of Cardiology, West China Hospital, Sichuan University/West China School of Nursing, Sichuan University, Chengdu, China; ^2^Department of Infrastructure, West China Hospital, Sichuan University, Chengdu, China; ^3^Department of Neurology, West China Hospital, Sichuan University/West China School of Nursing, Sichuan University, Chengdu, China; ^4^Department of Radiology, West China Hospital, Sichuan University/West China School of Nursing, Sichuan University, Chengdu, China

**Keywords:** caregivers for hospitalized stroke survivors, resilience, uncertainty in illness, caregiving ability, structural equation modeling

## Abstract

**Background:**

In China, stroke survivors are usually cared for by their family members. However, the caregiving ability of these informal caregivers remain inadequate during the hospitalization of their family members following a sudden onset of a stroke, and this sudden need for care overwhelms caregivers even after the hospital discharge. Therefore, research is required to identify predictors of caregiving ability that could be targeted in future interventions aimed at improving caregiving skills and reducing the burden on caregivers who care for stroke survivors.

**Materials and methods:**

From August 2019 to February 2020, stroke survivors were hospitalized for the first time, and their family caregivers were registered *via* convenience sampling. Caregiver demographic information, resilience status, uncertainty in illness, caregiving ability, and patients' severity of stroke were measured using standardized questionnaires. Structural equation modeling was used to test the proposed model, where caregiver resilience and stroke severity predicted caregiving ability directly, and uncertainty in illness mediated the association between caregiver resilience and caregiving ability.

**Results:**

A total of 306 dyads were included in the study. The tested model fit the data well (χ^2^ = 118.2, df = 64, RMSEA = 0.053, CFI = 0.946, TLI = 0.923). Statistically significant pathways linked caregivers' resilience status to uncertainty in illness (β = −0.558, S.E. = 0.022, *P* < 0.01), caregivers' resilience to the status of caregiving ability (β = −0.269, S.E. = 0.013, *P* < 0.01) and caregivers' uncertainty about the illness to caregiving ability (β = 0.687, S.E. = 0.051, *P* < 0.01). We also found that caregivers' uncertainty in illness mediated the association between caregivers' resilience and caregiving ability (β = −0.384, S.E. = 0.061, *P* < 0.01).

**Conclusions:**

Our structural equation modeling result identified resilience and uncertainty about the illness as predictors of the caregiving ability of informal family caregivers who suffered from care burdens. Supporting family caregivers to build their resilience and reduce illness uncertainty may improve caregiving for stroke survivors.

## Introduction

Stroke is a common vascular disease. Based on the Global Burden of Disease (GBD) reports, stroke may result in a sharp increase in disease burden, especially in developing countries (1). Even though a decline in stroke incidence has been observed, it remains the second most common cause of death and the third most common cause of disability worldwide (2). Globally, the aging of the population and accumulated risk factors increase the lifetime risk of stroke. According to the Brief Report on Stroke Prevention and Treatment in China, there is an obvious rising trend of disability-adjusted life years (DALYs) related to ischemic stroke (3). A 5-year nationwide study revealed that 45% of Chinese post-stroke patients had a disability (4), which poses a big challenge to society.

In China, family members mainly take on the responsibility of caring for stroke survivors in hospitals, communities, or at home. This is not only due to the traditional Chinese family culture, in which close and affectionate relationships bind family members to the dependent person (5, 6) but also because people tend to shorten their hospital stay or cannot afford the expense of professional rehabilitation institutions (7). However, this poses a huge problem for informal caregivers who are not medically trained in caregiving skills. They feel that their lives have been entirely disrupted, like “turned their lives upside down” (8). This stress and burden also put the informal caregivers of people with stroke at an increased risk of physiological comorbidity, isolation, and anxiety, resulting in a poor quality of life after long-term home care (9–11). They will likely end up as “the second patient” in the family if the care burden is not properly managed (12). Varying stroke severity results in a large variety of patient outcomes, ranging from full recovery to severe disability or death. Disabled stroke survivors who cannot complete their daily lives independently must rely on their caregivers, such as nurses, nursing workers, or family members. Caregiving ability are regarded as the ability to complete caregiving tasks, and it involves three components: dealing with activities of daily living, intrapersonal tasks, and interpersonal ties (13). It is rational to assume that increasing the stroke severity would reduce caregivers' health-related quality of life and have a negative impact on their caregiving ability in the long term (14).

The dynamic nature of resilience means that it can serve as an asset to those who possess it. It regulates the impact of adverse life events (15, 16) and the recovery from negative events, e.g., more resilient caregivers may suffer less stress. Resilience, in other words, is an inherent resource that broadens a person's scope of thinking and helps them deal with adversities or challenges to avoid distress. Caregivers of people with stroke exhibit resilience by adopting different coping and behavioral strategies in the face of adversity, which may indirectly enhance their caregiving ability (17, 18). To the best of our knowledge, no research has investigated the direct relationship between resilience and the caregiving ability of caregivers of people with stroke, but it has been reported that caregivers' resilience plays an important role in reducing care burden, possibly improving their caregiving ability (19).

Uncertainty in illnesses makes it difficult for decision-makers to identify the illness-related causes due to insufficient cues (20). After experiencing a stroke, patients and their family members are thrust into unfamiliar positions, resulting in huge uncertainty, anxiety, and even hopelessness about the patients' recovery (21). The understanding of caregivers about the disease may significantly impact their caregiving ability (22). If caregivers are unable to deal with the stress and uncertainty surrounding the diseases, it inevitably undermines confidence in their caregiving ability (23). On the contrary, Swallow et al. reported that uncertainty in illness pushes caregivers to improve their caregiving skills (24). With regard to the association between resilience and uncertainty surrounding the illness, resilient people are always in a state of mind to acknowledge the complexity of life and embrace the changes and uncertainty (25); however, we have not found any research discussing the association between resilience and uncertainty in illness in the context of caregiving ability yet.

**Figure 1:**
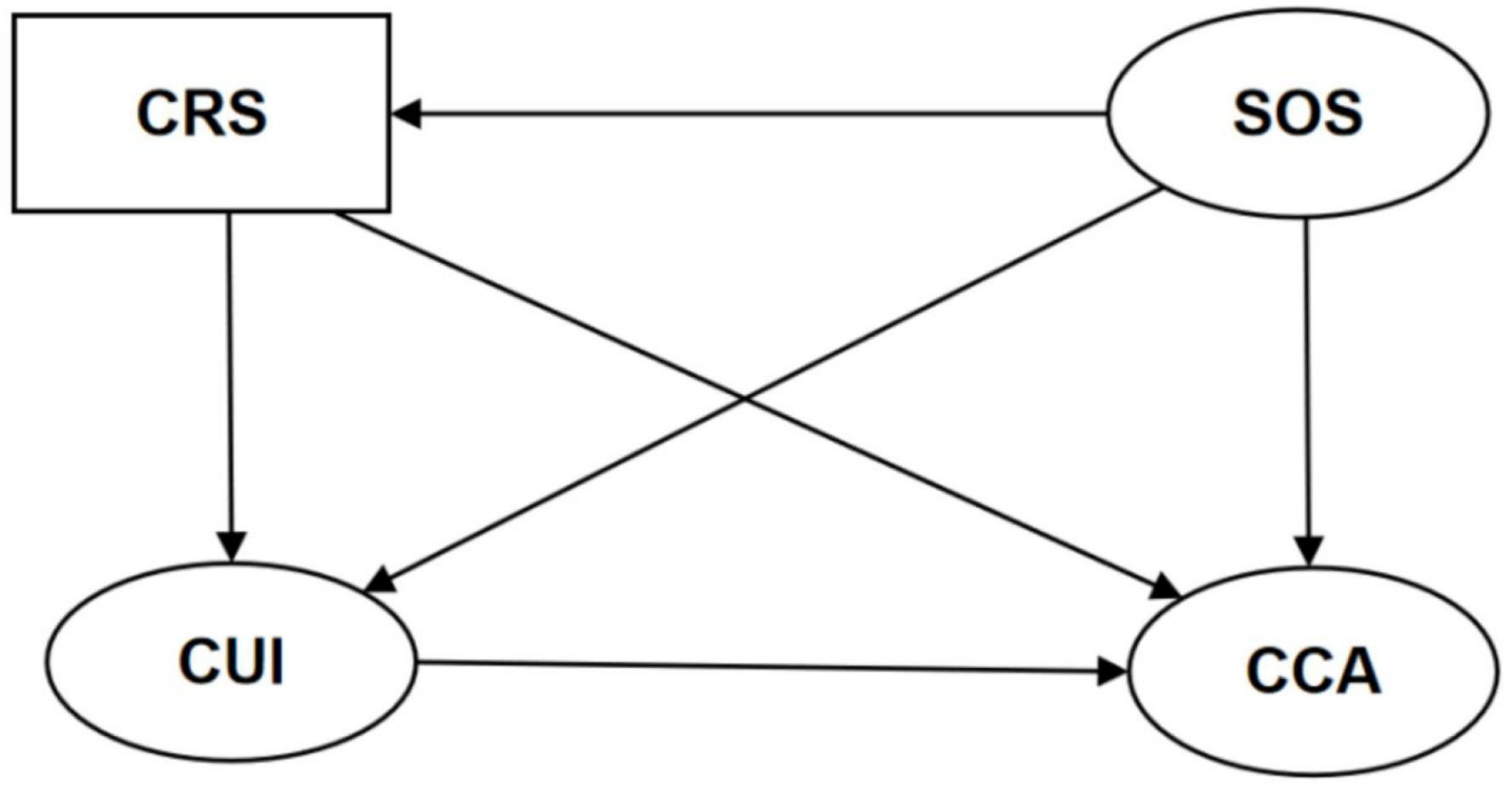
The hypothetical model. CRS, caregivers' resilience state; SOS, the severity of stroke; CUI, caregivers' uncertainty about the illness; CCA, caregivers' caregiving ability.

The caregiving skills of caregivers are inadequate, especially after the first stroke. Identifying predictors of caregiving ability can provide insight into how caregivers can be supported to reduce the burden and improve their caregiving skills. Given the lack of previous research on the associations between resilience, uncertainty surrounding illness, and caregiving ability, we aimed to explore the relationship between these three variables in the caregivers of people with stroke. Based on the literature review, we hypothesized that (1) resilience might negatively predict uncertainty in the illness of caregivers of people with stroke; (2) uncertainty surrounding the illness may negatively predict the caregiving ability of caregivers of people with stroke; (3) resilience may positively predict the caregiving ability of caregivers of people with stroke; (4) caregivers' resilience may indirectly predict their caregiving ability through the mediating effect of uncertainty about the illness; (5) stroke severity may indirectly predict caregivers' caregiving ability through the mediating role of uncertainty about the illness; and (6) stroke severity may indirectly predict caregivers' caregiving ability through the mediating role of caregivers' resilience state, as shown in Figure 1.

## Materials and methods

### Design and participants

In this study, we employed a cross-sectional study design with convenience sampling. We recruited a stroke survivor-caregiver dyad, and the stroke patient's inclusion criteria were as follows: (1) stroke survivors who were hospitalized for the first time, and (2) people whose daily lives were normal before the onset of stroke but were partially or completely impaired after the onset. Conversely, stroke survivors with mental health disorders or who died after hospitalization were excluded.

The inclusion criteria for caregivers with stroke were as follows: (1) one of the relatives of stroke survivors or the main caregiver was appointed by the patient if there were several caregivers; (2) the caregiving time was over 1 week and more than 4 h/day; (3) age ≥18 years; and (4) no mental disorders and severe cognitive impairment. Data were collected from August 2019 to February 2020 at West China Hospital (Chengdu, China). The eligible volunteers (including stroke survivors and their caregivers) completed the self-reporting questionnaires on the day of discharge in a quiet environment. Questionnaires were collected immediately after completion, and uniformly trained researchers checked the data quality. A total of 320 eligible stroke survivor-caregiver couples were recruited. Fourteen couples either refused to participate or withdrew from the study. Finally, 306 couples were included (response rate of 95.6%).

### Measurements

#### The demographic information of family caregivers

The demographic data of stroke survivors and their caregivers were collected using an information questionnaire developed by consultants (a multidisciplinary management team for stroke patients in the neurology department). The questionnaire contained caregivers' gender, age, type, educational level, employment status, residential district, marital status, mean household incomes per month, self-perceived health condition, and similar caregiving experiences. It also contains the type of stroke and the location of the lesion in the patients' brains.

#### Resilience level of family caregivers

Caregiver resilience was measured using the Chinese version of the 10-item Connor-Davidson Resilience Scale (CD-RISC-10) (26, 27). It is a 5-point rating scale that only has one dimension, ranging from 0 (“never”) to 4 (“almost always”); the total score is the sum of the points of all items (range 0–40). A high total score indicates a high level of resilience. The Cronbach's α of this scale in our study was 0.958.

#### Uncertainty in illness among family caregivers

Illness uncertainty was measured using the 30-item Chinese version of the Mishel Uncertainty in Illness Scale for Family Members (MUIS-FM) (28). It measured the uncertainty of an individual unable to determine the meaning of illness-related events (29). The Chinese version of MUIS-FM is a 5-point Likert rating scale consisting of four dimensions: ambiguity, complexity, unpredictability, and lack of information. Each item was scored from 1 (strongly disagree) to 5 (strongly agree). Items 6, 9, 11, 19, 23, 25, 27, 28, 29, and 30 were scored using a reverse point. Total sum scores range from 30 to 150. A higher score indicated greater uncertainty surrounding the illness. The Cronbach's α in our study was 0.825.

#### Caregiving ability among family caregivers

The caregiving ability of family caregivers were measured using the Chinese version of the Family Caregiver Task Inventory (FCTI) (13, 30). The instrument consists of 25 items with an answering scale of 0 (not difficult) to 2 (extremely difficult), and the total scores range from 0 to 50. The FCTI is composed of 5 dimensions: “learning to cope with a new role,” “providing care with the care-receiver's needs in mind,” “managing one's own emotional needs,” “appraising supportive resources,” and “balancing caregiving needs and one's own needs.” The higher the total score, the more difficult it is for caregivers to take care of patients. The Cronbach's α of the Chinese version of FCTI in our study was 0.876.

#### Stroke severity of stroke survivors

We applied the Barthel Index and the National Institute of Health Stroke Scale to measure stroke severity. The Barthel Index (BI) is a 10-item scale that measures a person's ability to perform activities of daily living (ADL) (31). Total scores range from 0 (worst mobility in ADL) to 100 (full mobility in ADL). A higher total score indicates greater independence in ADL. The Barthel Index assesses the patient's ability by completing the following tasks: eating, bathing, grooming, dressing, bowel and bladder control, ability to use the toilet, chair/bed transferring, ambulation, and stair-climbing (32). The Cronbach's α in our study was 0.908. We treated the BI scale as a numerical scale rather than categorizing it.

The National Institute of Health Stroke Scale (NIHSS) is a 15-item instrument originally developed in 1989 (33), and it is a recommended tool for evaluating stroke severity. The total score is the sum of all the items and ranges from 0 to 42. A higher score reflects greater stroke severity. The NIHSS includes the following nine domains: level of consciousness, eye movements, the integrity of visual fields, facial movements, muscle strength of arms and legs, sensation, coordination, language, speech, and neglect (34). The Cronbach's α in our study was 0.883.

### Ethical approval

Our study procedures were approved by the ethics committee of West China Hospital (2018 Review No. 27). All the included participants gave written informed consent. Confidentiality and anonymity were maintained during the study, and our members interpreted research procedure-related questions. Participants were allowed to withdraw whenever they no longer wanted to participate in the study (35).

### Statistical analysis

The demographics of the first-ever hospitalized stroke survivors and their caregivers were summarized by descriptive statistics, including means, standard deviations, frequencies, and percentages. The correlation between resilience, uncertainty in illness, caregivers' caregiving ability, and stroke severity (measured by NIHSS scores and BI scores) was analyzed by Pearson correlation analysis. An independent-sample *t*-test and a one-way ANOVA were conducted to identify sociodemographic predictors of the main variables of interest (i.e., resilience, uncertainty in illness, and caregiving ability).

**Figure 2:**
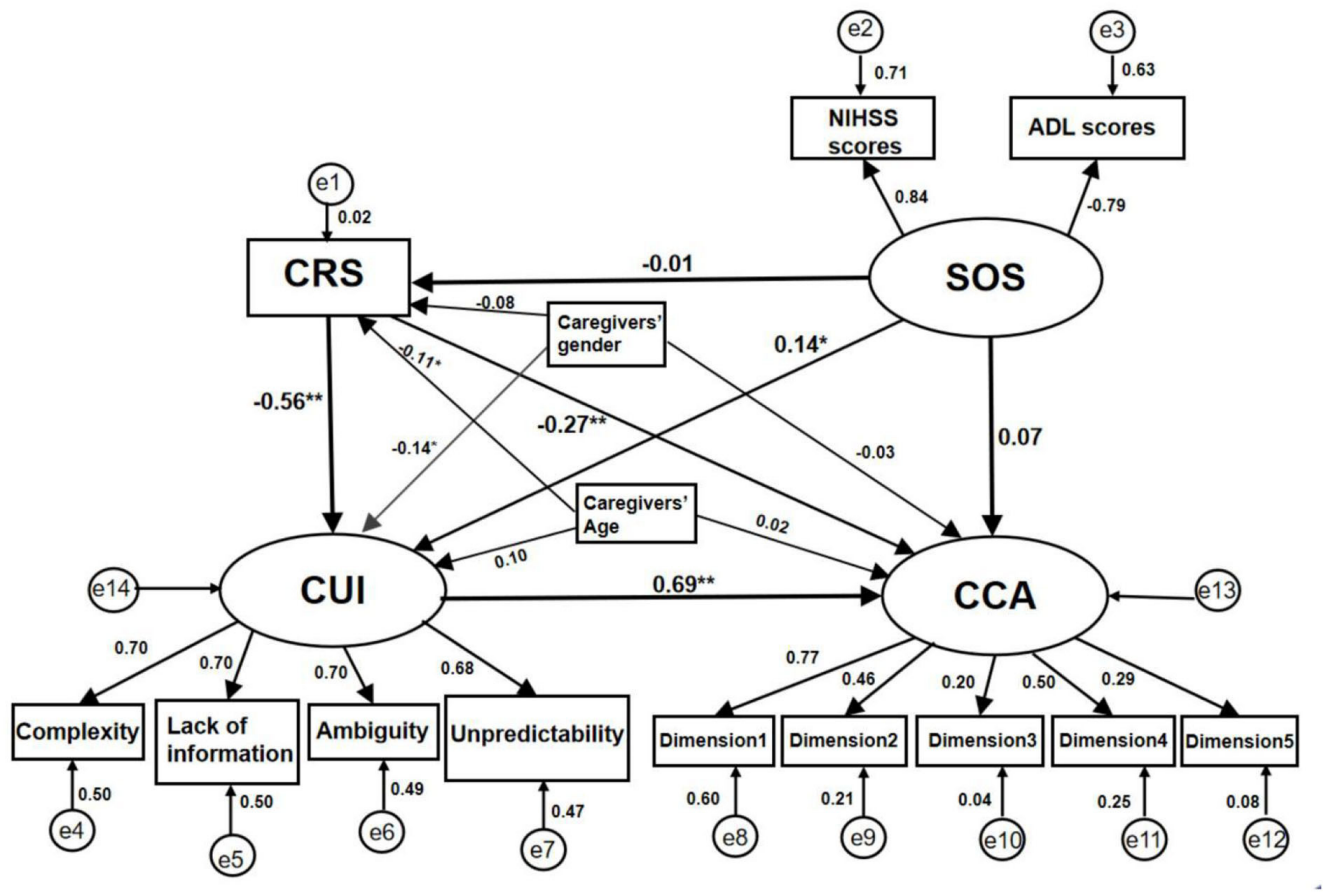
Standardization coefficient of the SEM in the caregivers of people with stroke. CRS, caregivers' resilience state; SOS, the severity of stroke; CUI, caregivers' uncertainty in illness; CCA, caregivers' caregiving ability; Dimension 1 = Learning to cope with a new role; Dimension 2 = Providing care with the care-receiver's needs in mind; Dimension 3 = Managing one's own emotional needs; Dimension 4 = Appraising supportive resources; Dimension 5 = Balancing caregiving needs and one's own needs; *P < 0.05; **P < 0.01.

A structural equation model (SEM) was used to test the hypothesized associations between the patient's severity of stroke and their caregivers' resilience, uncertainty in illness, and caregiving ability. Latent variables, observed variables, and residual errors were represented by ovals, rectangles, and circles, respectively. The three latent variables were stroke severity (SOS), caregivers' uncertainty about the illness (CUI), and caregivers' caregiving ability (CCA). Caregivers' resilience state (CRS), gender, and age were the observed variables. All dimensions of the MUIS-FM scale and the FCTI scale were considered observed variables for CUI and CCA. The model examined whether caregivers' resilience predicted caregiving ability directly and indirectly *via* uncertainty in illness while considering caregiver age, sex, and the stroke severity experienced by the stroke survivors (see Figure 2).

SEM was used to verify the hypothetical model. The χ^2^ statistic, comparative fit index (CFI), root mean square error of approximation (RMSEA), and Tucker-Lewis fit index (TLI) were performed to estimate the extent to which the model reproduced the empirical covariance matrix of the involved variables. A CFI > 0.9, a TLI > 0.9, and an RMSEA < 0.08 were regarded as good model fits (36–39). Bootstrapping evaluated the significance of direct, indirect, and total effects between the measured factors with a bootstrap sample of 1,000 (40). SPSS version 22.0 (IBM Corporation, Armonk, NY, USA) and Amos version 21.0 (IBM Corporation, Armonk, NY, USA) were used in this study, and a *P* ≤ 0.05 was considered statistically significant.

## Results

### Sociodemographic characteristics of the samples

In our study, 306 eligible stroke survivor-caregiver dyads were recruited, and the demographic characteristics of caregivers are shown in Table 1. The mean age of stroke survivors was 64.09 ± 13.90 years, and the mean age of their caregivers was 50.78 ± 13.19 years. 223 (72.9%) patients had ≥1 comorbidity associated with stroke. A total of 157 (51.3%) patients had a BI score of <40, and only 54 (17.6%) patients had a family history of stroke. The patients' spouses became the caregivers in most cases (48%), while the patients' offspring became the caregivers in 39.5% of the cases. The remaining caregivers were patients' daughters-in-law, sons-in-law (3.3%), and other relatives (9.2%).

**Table 1:** Univariate analysis of sociodemographic characteristics of caregivers and the measured variables (*n* = 306).

**Variables**	***N*** **(%)**	**Resilience** ^**a**^		**Uncertainty in illness** ^**b**^		**Caregiving ability** ^**c**^	
		**M ±SD**	***t/F***	**M ±SD**	***t/F***	**M ±SD**	***t/F***
**Age(y)**							
≤44	94 (30.7)	29.93 ± 5.59	3.475^*^	69.33 ± 12.35	3.714^*^	5.88 ± 3.77	0.866
45–64	164 (53.6)	28.01 ± 5.83		70.65 ± 13.09		6.32 ± 3.49	
≥65	48 (15.7)	28.04 ± 6.33		75.52 ± 14.15		6.71 ± 4.13	
**Gender**							
Male	101 (33)	29.28 ± 5.71	0.581	72.42 ± 13.66	0.963	6.31 ± 3.94	0.211
Female	205 (67)	28.27 ± 5.95		70.31 ± 12.89		6.21 ± 3.55	
**Educational level**							
Elementary school or less	79 (25.8)	26.49 ± 6.36	11.75^**^	75.75 ± 14.05	5.46^**^	7.75 ± 4.11	9.22^**^
Middle school	97 (31.7)	27.87 ± 5.18		69.21 ± 12.79		6.38 ± 3.38	
High school	55 (18)	28.78 ± 5.89		71.31 ± 11.82		5.82 ± 3.32	
College or higher	75 (24.5)	31.65 ± 5.0		68.12 ± 12.45		4.8 ± 3.23	
**Marital status**							
With a partner	288 (94.1)	28.59 ± 5.95	2.327	70.72 ± 13.26	1.055	6.18 ± 3.59	2.316
Without a partner	18 (5.9)	28.83 ± 4.79		75.61 ± 10.62		7.22 ± 4.88	
**Monthly household income (RMB)**							
≤1,000	52 (16.9)	28.25 ± 5.89	5.871^**^	69.5 ± 14.01	9.218^**^	6.48 ± 3.55	5.871^**^
1,001–3,000	92 (30.1)	26.86 ± 5.82		75 ± 13.33		7.61 ± 3.98	
3,001–6,000	88 (28.8)	29 ± 5.98		71.9 ± 11.71		5.93 ± 3.45	
≥6,001	74 (24.2)	30.55 ± 5.26		66.04 ± 12.39		4.76 ± 3.02	
**Employment status**							
On job	148 (48.4)	29.01 ± 5.66	1.325	69.18 ± 12.31	2.247	5.67 ± 3.33	5.306^**^
Unemployed	88 (28.7)	28.85 ± 6.28		72.83 ± 13.89		6.07 ± 3.79	
Retired	59 (19.3)	27.25 ± 5.96		71.85 ± 13.92		7.53 ± 3.58	
Other	11 (3.6)	28.45 ± 4.80		76.45 ± 11.73		8.55 ± 5.5	
**Residential district**							
City	185 (60.5)	29.05 ± 5.69	0.6	70.43 ± 12.99	0.258	6.01 ± 3.59	0.648
Rural area	121 (39.5)	27.93 ± 6.12		71.88 ± 13.42		6.61 ± 3.79	
**Self-perceived health status**							
Very good	57 (18.6)	29.72 ± 5.11	3.984^**^	68.96 ± 13.52	*2.594*	5.84 ± 3.83	*5.028* ^**^
Good	147 (48)	29.20 ± 5.51		70.37 ± 12.06		5.63 ± 3.18	
Not bad	77 (25.2)	26.70 ± 6.44		74.44 ± 14.95		7.47 ± 4.29	
Poor	25 (8.2)	28.44 ± 6.79		68.80 ± 11.27		7.04 ± 3.16	
**Previous similar care experience**							
Yes	110 (35.9)	28.39 ± 6.30	−0.475	71.18 ± 13.03	0.174	6.18 ± 3.39	−0.225
No	196 (64.1)	28.72 ± 5.65		70.91 ± 13.27		6.28 ± 3.84	

* *P* < 0.05;

** *P* < 0.01.

a The resilience of caregivers was measured with the Connor-Davidson Resilience Scale 10-item form (CD-RISC-10).

b Uncertainty in illness of caregivers was measured with the Mishel Uncertainty in illness Scale-Family Member Form (MUIS-FM).

c Caregiving ability of caregivers were measured with the Family Caregiver Task Inventory (FCTI).

### Sociodemographic and health-related predictors of resilience, uncertainty in illness, and caregiving ability of caregivers

Lower education levels and household incomes were associated with lower caregivers' resilience, greater uncertainty in illness, and poorer caregiving ability. Poorer self-perceived health status predicted lower caregivers' resilience and poorer caregiving ability. The older the caregivers, the higher the uncertainty about the illness and the lower the resilience (see Table 1).

### Correlations between resilience, uncertainty in illness, caregiving ability of caregivers, and severity of stroke

We found that the total scores of the 10-item Connor-Davidson Resilience Scale (CD-RISC-10), the Mishel Uncertainty in Illness Scale for Family Members (MUIS-FM), the Family Caregiver Task Inventory (FCTI) scale, and the Barthel Index (BI) scale were approximately distributed normally, except for the total scores of the National Institute of Health Stroke Scale (NIHSS). The mean total scores of the CD-RISC-10 scale, the MUIS-FM scale, the FCTI scale, the BI scale, and the NIHSS scale among caregivers were 28.6 ± 5.88, 71.01 ± 13.16, 6.25 ± 3.68, 43.94 ± 29.16, and 7.04 ± 6.46, respectively. Higher resilience of caregivers was associated with lower uncertainty about the illness (r = −0.493, *P* < 0.01) and better caregiving ability (r = −0.5, *P* < 0.01); lower uncertainty about the illness of caregivers predicted better caregiving ability (r = 0.585, *P* < 0.01). In addition, the worse the caregivers' ability to care (see Table 2).

**Table 2:** Correlations matrix of the measured variables.

**Variables**	**M ±SD**	**1**	**2**	**3**	**4**	**5**
1. Resilience	28.6 ± 5.88	1				
2. Uncertainty in illness	71.01 ± 13.16	−0.493^**^	1			
3. Caregiving ability	6.25 ± 3.68	−0.5^**^	0.585^**^	1		
4. BI	43.94 ± 29.16	−0.066	−0.094	−0.126^*^	1	
5. NIHSS	7.04 ± 6.46	−0.035	0.132^*^	0.163^**^	−0.638^**^	1

BI, the Barthel Index; NIHSS, National Institute of Health Stroke Scale; M ± SD, mean ± standard deviation;

* *P* < 0.05;

** *P* < 0.01.

### Analyzing parameter estimation and verifying the suitability of the hypothetical model

The standardized SEM diagram of some sociodemographic factors, caregivers' resilience, uncertainty in illness, their caregiving ability, and the stroke severity experienced by the stroke survivors are shown in Figure 2. Based on the regression and correlation paths of the analyzed model, we discovered six beta path coefficients that were statistically significant. There was caregivers' resilience status to uncertainty about the illness (β = −0.558, S.E. = 0.022, *P* < 0.01), caregivers' resilience status to caregiving ability (β = −0.269, S.E. = 0.013, *P* < 0.01), uncertainty about the illness to caregiving ability (β = 0.687, S.E. = 0.051, *P* < 0.01), the stroke severity to caregivers' uncertainty about the illness (β = 0.140, S.E. = 0.026, *P* < 0.05), caregivers' gender to uncertainty about the illness (β = −0.138, S.E. = 0.221, *P* < 0.05), and caregivers' age to caregivers' resilience status (β = −0.105, S.E. = 0.026, *P* < 0.05). Eventually, the fit indices displayed a good model fit to the data with χ^2^ = 118.2, df = 64, RMSEA = 0.053, CFI = 0.946, and TLI = 0.923.

The bootstrapped 95% confidence interval confirmed the existence of total, direct, and indirect effects. Direct associations were found between CRS and CUI (β = −0.558, S.E. = 0.050, *P* < 0.01), CRS and CCA (β = −0.269, S.E. = 0.079, *P* < 0.01), SOS and CUI (β = 0.140, S.E. = 0.070, *P* < 0.05), CUI and CCA (β = 0.687, S.E. = 0.079, *P* < 0.01). Caregivers' uncertainty about the illness was a mediator between their resilience status and caregiving ability (β = −0.384, S.E. = 0.061, *P* < 0.01). The standardized direct, indirect, and total effects are listed in Table 3.

**Table 3:** The standardized total, indirect and direct effects of the adjusted hypothetical model.

**Variables**	**Standardized** **estimate**	**S.E**.	**C.R**.	***P*** **-value**	**95% CI of the total** **effect**		**95% CI of the direct** **effect**		**95% CI of the indirect** **effect**	
					**Lower**	**Upper**	**Lower**	**Upper**	**Lower**	**Upper**
SOS → CRS	−0.010	0.086	−0.127	0.995	−0.141	0.167	−0.141	0.167		
SOS → CUI	0.140	0.026	1.882	0.023	−0.011	0.307	(0.018, 0.283)^*^		−0.096	0.076
SOS → CCA	0.066	0.012	1.103	0.188	−0.001	0.353	−0.035	0.213	−0.043	0.234
CRS → CUI	−0.558	0.022	−8.331	0.001	(−0.656, −0.462)^**^		(−0.656, −0.462)^**^			
CRS → CCA	−0.269	0.013	−3.926	0.002	(−0.772, −0.541)^**^		(−0.415, −0.104)^**^		(−0.522, −0.278)^**^	
CUI → CCA	0.687	0.051	7.619	0.002	(0.546, 0.853)^**^		(0.546, 0.853)^**^			

SOS, severity of stroke; CRS, Caregivers' resilience status; CUI, Caregivers' uncertainty in illness; CCA, Caregivers' caregiving ability; C.R., Critical ratio; S.E., Standard error; CI, confidence interval;

* *P* < 0.05;

** *P* < 0.01.

## Discussion

Family support is a key resource for sick patients in China, but caregivers without professional training often find it difficult to help stroke survivors, especially those unable to accomplish activities of daily living (30). This study identified the associations between sociodemographic factors, resilience, uncertainty about the illness, and caregiving skills. As hypothesized, the severity of strokes, caregiver resilience, and caregiver uncertainty about the illness predicted the caregiving ability of informal family caregivers, and uncertainty about the illness was found to mediate the relationship between caregiver resilience and their caregiving ability. These findings could inform future studies aiming to improve the caregiving ability of caregivers.

In this study, the total resilience score was at an intermediate level, consistent with studies on informal caregivers of cancer patients (41–43). Half of our hospitalized stroke patients showed a low ability to perform activities of daily living, and their caregivers were expected to take on more care tasks. Our caregivers could get convenient and timely help from professional medical staff when patients were in the hospital. Hence, their caregiving ability were better than those of caregivers who cared for the disabled, older people in the community (6.25 vs. 11.2) (44). Uncertainty in the illness of caregivers of people with stroke in our study was lower than in other caregiver populations (71.01 vs. 83.73) (45). This may be because caregivers of older patients or patients with recurrent strokes experienced more uncertain situations.

We found that caregivers showed a higher level of resilience and less uncertainty about the illness when they were younger. Older caregivers may be frustrated by poor physical health and probably have reduced access to high income, which is, in part, linked to lower individual resilience and uncertainty (46–52). Our findings also revealed that caregivers with a good self-perceived health status were more likely to report better caregiving ability and resilience. In other words, caregivers with poorer self-perceived health might have more physiological or psychological problems. They may be unable to improve their caregiving ability by seeking more relevant knowledge or help (53).

Moreover, less resilient caregivers may pay less attention to their physical and mental health (48). In our study, educational level and household income were associated with caregivers' resilience, uncertainty about the illness, and caregiving ability. Caregivers with a lower level of education may have limited access to resources to improve their caregiving ability, which may result in numerous problems: being misled by wrong advice from laypeople due to inadequate knowledge, readmissions due to complications, and being likely to take up underpaid jobs due to societal pressure (47, 54). Families with lower incomes may experience financial stress and more uncertainty, affecting caregivers' caregiving ability (46). On the contrary, educated individuals who are confident in their resilience may think more positively about adversities and may have more opportunities to receive support, so their caregiving ability probably improve even in the face of emerging care burdens (51, 52, 55).

In summary, resilience and uncertainty during an illness may partially mediate associations between educational level or household income and caregiving ability. Sociodemographic characteristics of caregivers may also moderate associations between resilience and caregiving ability. Our model did not test these pathways and could be explored in future research.

We confirmed that lower caregivers' uncertainty about the illness and higher resilience were related to better caregiving ability, while lower resilience and more severe stroke were associated with higher uncertainty in the illness of caregivers of people with stroke. Caregivers who do not understand their new role well suffer from uncertainty about the present and future in the initial caregiving days. These caregivers often lack specific knowledge about the dos and don'ts for stroke survivors, which may significantly influence the recovery time and treatment outcomes (56, 57). Byun et al. stated that caregivers' uncertainty about stroke survivors' outcomes might be heightened in the early poststroke period because they were unsure of the extent of damage to the physical or cognitive function of the patients (58, 59). A severe stroke (partially reflected by NIHSS and BI scores) is accompanied by a strong sense of uncertainty, which predicts poorer caregivers' caregiving ability, as greater physical disability among patients leads to an increased care burden and a poorer quality of life for caregivers (60). Stroke survivors require collaborative care management, including hospital, community, and family involvement, as stroke recovery is long and arduous. The caregiving ability of family caregivers have been reported to deteriorate when they oversee complex and demanding tasks (61). Discharge from the hospital generally limits access to professional treatment and nursing. However, this discharge-related stress may be significantly reduced if patients and caregivers are provided with more information, guidance, and caregiving training by professionals and other wardmates (62). An inpatient training plan may prepare patients and caregivers to deal with the transfer from hospitals to communities or homes; a better option may be integrating home-based and community-based caregiving training plans to improve caregivers' caregiving ability (63, 64).

The effect of resilience, which consists of individual, social, organizational, and structural aspects, cannot be ignored. More resilience resources may improve individuals' caregiving ability and boost their confidence (65–67). In informal caregiving, caregivers with high resilience tend to experience a low care burden even in the presence of high care demand from patients (23, 68). More resilient caregivers always exert more active coping toward preparing for and executing caregiving tasks, which is good for improving their caregiving ability (69).

We also found a significant negative correlation between resilience and uncertainty about the illness. It was reported that providing stroke-related information and skills training could improve caregivers' resilience, which was considered a key construct to regulate future uncertainties (70, 71). On the other hand, Hetra et al. reported that coping with uncertainty was more likely to foster resilience after experiencing life-threatening accidents, such as a sudden stroke (72). The resilience enhancement program emphasized community integration among caregivers of people with stroke by seeking emotional support and providing psychoeducation and information. Building esteem and networking could be a valuable intervention to improve their caregiving ability (73, 74).

The other finding from our study was that caregivers' resilience status yielded a significant partial indirect effect on their caregiving ability through uncertainty in illness. The sudden and unpredictable nature of stroke provides multiple ambiguous disease-related situations for patients and their caregivers, such as unanticipated disease recurrence and unreliable illness symptoms. Caregivers with more uncertainty about stroke diagnosis and prognosis are inclined to regard worse future outcomes as inevitable, resulting in ineffective coping strategies and poor caregiving ability (45, 75). Nevertheless, resilience is a dynamic personal disposition influenced by both genes and environment (76, 77), and it is associated with less negative emotions, higher confidence, optimism, and acceptance. The intrinsic characteristics of resilience (such as meaning in life, self-acceptance, perseverance, balance, and self-reliance) keep the hypothalamic-pituitary-adrenal axis normal. In other words, higher resilience prevents individuals from negative emotional outcomes (78, 79). This process may help individuals successfully adapt to stroke-related uncertainty or hardship, which makes it possible to improve the caregiving ability of caregivers of people with stroke.

## Limitations

Our study has several limitations. Firstly, the conclusive statements about causality in the SEM should be interpreted cautiously, as the study had a single-center cross-sectional design and lacked temporality and direction. These results may not apply to all types of caregivers of people with stroke, such as those of patients with recurrent stroke. Multi-center and longitudinal studies are recommended to confirm our findings. Secondly, self-reporting bias might affect the results, especially in the caregiving ability assessment (80). Therefore, in the future, patients and healthcare providers will need to complete observation reports of measured variables. Thirdly, our SEM did not include all potential variables, and other variables that could affect caregivers' caregiving ability were not thoroughly examined. Consequently, future studies should investigate other mediating variables to fully understand the complex relationship between psychosocial factors and the caregiving ability of caregivers of people with stroke.

## Conclusions

To the best of our knowledge, this study is the first to explore the relationships between stroke severity, demographic factors (caregivers' age and gender), psychosocial health-related factors (resilience, uncertainty in illness), and the caregiving ability of caregivers who cared for hospitalized stroke survivors. Our hypothetical model identified caregivers' resilience and uncertainty about the illness as predictors of the caregiving ability of informal family caregivers who lack professional training. It paves the way for future structured interventions using psychoeducation. Supporting family caregivers to build their inner resilience may help to improve caregiving for stroke survivors. The mediating role of uncertainty about an illness should also be considered simultaneously as a potential pathway for interventions.

## Data availability statement

The raw data supporting the conclusions of this article will be made available by the authors, without undue reservation.

## Ethics statement

The studies involving human participants were reviewed and approved by Ethics Committee of West China Hospital, Sichuan University. The patients/participants provided their written informed consent to participate in this study.

## Author contributions

LZ and JW conceived and designed the study and they had full access to all the data of this research. JC and ST took responsibility for the data collection. JW and JC drafted the manuscript. JC and RY had revised the manuscript carefully to keep the accuracy. All authors had reviewed and approved the final version of the manuscript.

## Funding

The research was funded by West China Nursing Discipline Development Special Fund Project, Sichuan University (Grant Nos. HXHL21031, HXHL20004, and HXHL19004).

## Conflict of interest

The authors declare that the research was conducted in the absence of any commercial or financial relationships that could be construed as a potential conflict of interest.

## Publisher's note

All claims expressed in this article are solely those of the authors and do not necessarily represent those of their affiliated organizations, or those of the publisher, the editors and the reviewers. Any product that may be evaluated in this article, or claim that may be made by its manufacturer, is not guaranteed or endorsed by the publisher.
