# Corrigendum: Resilience and caregiving ability among caregivers of people with stroke: the mediating role of uncertainty in illness

**DOI:** 10.3389/fpsyt.2023.1335672

**Published:** 2023-11-27

**Authors:** Jinyao Wang, Jun Cui, Shuangyan Tu, Rong Yang, Lihong Zhao

**Affiliations:** ^1^Department of Cardiology, West China Hospital, Sichuan University/West China School of Nursing, Sichuan University, Chengdu, China; ^2^Department of Infrastructure, West China Hospital, Sichuan University, Chengdu, China; ^3^Department of Neurology, West China Hospital, Sichuan University/West China School of Nursing, Sichuan University, Chengdu, China; ^4^Department of Radiology, West China Hospital, Sichuan University/West China School of Nursing, Sichuan University, Chengdu, China

**Keywords:** caregivers for hospitalized stroke survivors, resilience, uncertainty in illness, caregiving ability, structural equation modeling

In the published article, there was an error in affiliation Number 1. Instead of “West China School of Nursing, Sichuan University, Chengdu, China”, it should be “Department of Cardiology, West China Hospital, Sichuan University/West China School of Nursing, Sichuan University, Chengdu, China”.

In the published article, there was an error in affiliation Number 2. Instead of “Department of Cardiology, West China Hospital, Sichuan University, Chengdu, China”, it should be “Department of Infrastructure, West China Hospital, Sichuan University, Chengdu, China”.

In the published article, there was an error in affiliation Number 3. Instead of “West China Hospital, Sichuan University, Chengdu, China”, it should be “Department of Neurology, West China Hospital, Sichuan University/West China School of Nursing, Sichuan University, Chengdu, China”.

In the published article, there was an error in affiliation Number 4. Instead of “Department of Neurology, West China Hospital, Sichuan University, Chengdu, China”, it should be “Department of Radiology, West China Hospital, Sichuan University/West China School of Nursing, Sichuan University, Chengdu, China”.

The authors apologize for this error and state that this does not change the scientific conclusions of the article in any way. The original article has been updated.

